# Case of hyperpigmentation due to excessive androgen in a child

**DOI:** 10.11604/pamj.2023.45.118.34986

**Published:** 2023-07-07

**Authors:** Archana Thaware, Renu Rathi

**Affiliations:** 1 Department of Kaumarbhritya, Mahatma Gandhi Ayurved College Hospital and Research Centre, Datta Meghe Institute of Medical Sciences, Sawangi, Wardha, Maharashtra, India

**Keywords:** Hyper-androgen, hyperpigmentation, zonareticularis

## Image in medicine

During infancy and early childhood, low amounts of adrenal androgens are produced, and their secretion steadily rises with age, paralleling the expansion of the zonareticularis. The mechanism(s) through which this zone develops with age and how its secretion is regulated are not completely understood. Plasma concentrations of adrenal androgens rise throughout this process, while cortisol levels stay steady, implying that mechanisms other than corticotropin are at work. The mysterious androgen-stimulating factor could be one of them. The exact cause of premature pubarche is unknown. The early maturation of the zonareticularis, which leads to a rise in adrenal androgens to levels seen in early infancy, is thought to be the cause. It has also been suggested that an increase in androgen biosynthesis could be owing to the enzyme P450c17 being preferentially hyperphosphorylated due to an autosomal dominant activating mutation in the kinase responsible for the enzyme's serine/threonine phosphorylation. This is a rare case of hyper-androgen depicted in the figure with a hairy patch of hyperpigmentation.

**Figure 1:**
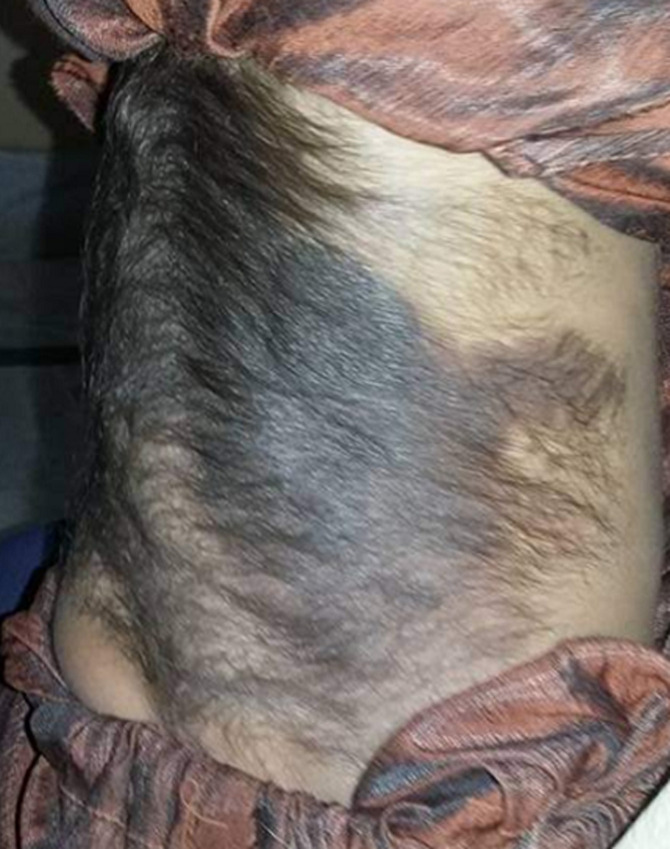
hairy patch of hyperpigmentation

